# CVD-grown monolayer MoS_2_ in bioabsorbable electronics and biosensors

**DOI:** 10.1038/s41467-018-03956-9

**Published:** 2018-04-27

**Authors:** Xiang Chen, Yong Ju Park, Minpyo Kang, Seung-Kyun Kang, Jahyun Koo, Sachin M. Shinde, Jiho Shin, Seunghyun Jeon, Gayoung Park, Ying Yan, Matthew R. MacEwan, Wilson Z. Ray, Kyung-Mi Lee, John A Rogers, Jong-Hyun Ahn

**Affiliations:** 1 School of Electrical and Electronic Engineering, Yonsei University, 50 Yonsei-ro, Seoul, 03722 Republic of Korea; 2 Department of Bio and Brain Engineering, KI for Health Science and Technology (KIHST), Korea Advanced Institute of Science and Technology, Daejeon, 34141 Republic of Korea; 3 Department of Materials Science and Engineering, Northwestern University, Evanston, 60208 IL USA; 4 Frederick Seitz Materials Research Laboratory, University of Illinois at Urbana-Champaign, Urbana, IL 61801 USA; 5 Department of Biochemistry and Molecular Biology, Korea University College of Medicine, Seoul, 02841 Republic of Korea; 6 Department of Neurological Surgery, Washington University School of Medicine, St Louis, MO 63110 USA; 7 Departments of Biomedical Engineering, Chemistry, Mechanical Engineering, Electrical Engineering and Computer Science, Center for Bio-Integrated Electronics, Simpson Querrey Institute for Nano/Biotechnology, Northwestern University, Evanston, IL 60208 USA

## Abstract

Transient electronics represents an emerging technology whose defining feature is an ability to dissolve, disintegrate or otherwise physically disappear in a controlled manner. Envisioned applications include resorbable/degradable biomedical implants, hardware-secure memory devices, and zero-impact environmental sensors. 2D materials may have essential roles in these systems due to their unique mechanical, thermal, electrical, and optical properties. Here, we study the bioabsorption of CVD-grown monolayer MoS_2_, including long-term cytotoxicity and immunological biocompatibility evaluations in biofluids and tissues of live animal models. The results show that MoS_2_ undergoes hydrolysis slowly in aqueous solutions without adverse biological effects. We also present a class of MoS_2_-based bioabsorbable and multi-functional sensor for intracranial monitoring of pressure, temperature, strain, and motion in animal models. Such technology offers specific, clinically relevant roles in diagnostic/therapeutic functions during recovery from traumatic brain injury. Our findings support the broader use of 2D materials in transient electronics and qualitatively expand the design options in other areas.

## Abstract (web summary)

Transient electronics entails the capability of electronic components to dissolve or reabsorb in a controlled manner when used in biomedical implants. Here, the authors perform a systematic study of the processes of hydrolysis, bioabsorption, cytotoxicity and immunological biocompatibility of monolayer MoS_2_.

## Introduction

Transient electronics is a class of technology defined by its ability to physically disappear or disintegrate in a controlled manner and/or at a specified time. Systems of this type can be designed to dissolve in water or biofluids, in a biocompatible and environmentally benign fashion, after a stable operating period^1,2^. The potential applications include medical diagnostic and therapeutic platforms that eliminate, through bioabsorption, any adverse long-term effects; environmental sensors that avoid the need for collection and recovery; and consumer devices that disintegrate with minimal processing costs and associated waste streams^1,2^. Recent reports describe bio/eco resorbable devices for use in transient photonics^3^, micromotors^4^, microsupercapacitors^5^, batteries^6^, triboelectric nanogenerators^7^, and diagnostics of electrical activity on the cerebral cortex^8^.

A critical challenge is in the development of constituent materials that are biocompatible and biodegradable^9^, yet also offer electronic grade properties as conductors, insulators, and semiconductors^1,2^. Recent work in this direction establishes some attractive options. Dissolvable metals include magnesium (Mg), iron (Fe), zinc (Zn), molybdenum (Mo), and tungsten (W)^10^. Dielectric polymers include poly(vinyl alcohol), polyvinylpyrrolidone, poly(lactic-co-glycolic acid) (PLGA), polylactic acid, and polycaprolactone, as well as biopolymers such as cellulose and silk. Such materials offer appealing attributes as substrates and encapsulation layers^11^. Inorganic dielectrics such as silicon dioxide (SiO_2_), silicon nitride (Si_3_N_4_), and magnesium oxide (MgO) can be used as interlayers and gate insulators. Choices for semiconductors include thin films or nanostructures of inorganic and organic materials, such as silicon (Si), germanium (Ge), Si-Ge, zinc oxide (ZnO), and 5,5′-bis-(7-dodecyl-9H-fluoren-2-yl)-2,2′-bithiophene (DDFTTF) and perylene diimide^12,13^.

The most sophisticated transient technologies use silicon nanomembranes (Si NMs), due to an advantageous combination of transport properties and nanoscale thicknesses, the latter of which allows their complete dissolution in water or biofluids due to hydrolysis over timescales that are relevant for degradation in the environment or the body^14^. A disadvantage is that the dissolution rates depend strongly on local chemistry, and can be strongly affected by the pH, the concentration, and type of ions and other factors. As a result, relatively thick Si NMs (>150 nm) are typically used to ensure operating times in the range of a few weeks^15^. Additional limitations of Si NMs follow from their brittle mechanical properties, with tensile failure strains of less than 1%, and their optical absorption throughout the visible range^16^. Therefore, developing transient semiconductors that simultaneously minimize, to a fundamental level, the total material content, and maximize the mechanical robustness, the electrical performance characteristics and the optical transparency represents an important direction for research in this emerging field.

Recent interest in two-dimensional (2D) transition-metal dichalcogenides derives from a set of unique electrical, optical, thermal, and mechanical properties that makes them attractive candidates as active materials in compact and lightweight integrated electronic systems^17,18^. Among these materials, molybdenum disulfide (MoS_2_) is one of the most important and extensively studied. In general, 2D MoS_2_ has several advantages^19^. First, the 2D confinement of electrons, especially in the case of monolayer MoS_2_, imparts ideal properties as a channel material for high-performance electronic or optoelectronic devices^20^. Second, its strong in-plane covalent bonding and atomic layer thickness yield excellent mechanical strength (breaking strain >2.2%), flexibility, and optical transparency; this collection of properties is important for applications in transparent, bendable devices^21^. High-throughput synthesis methods, such as those based on chemical vapor deposition (CVD) and metal-organic CVD, allow growth of large-scale MoS_2_ atomic layers and establishing pathways for their integration into practical systems^22–25^. Relevant properties will further be affected by its grain size due to the different defect densities associated with grain boundary and point defect^17,23^. Although many of these features are potentially crucial in transient electronics, little is known about the processes of biodegradation, in vivo and in vitro cytotoxicity, and immune responses associated with large-scale monolayer MoS_2_. In addition, preliminary work with exfoliated MoS_2_ nanosheets are not readily useful for this type of electronic devices and systems^26–29^.

Herein, we report the dissolution characteristics and behavior of both isolated crystals and continuous films of CVD-grown monolayer MoS_2_ in phosphate buffered saline (PBS) solutions at various temperatures and with different pH levels. Reducing the grain size of monolayer MoS_2_ and increasing the density of intrinsic defects increase the dissolution rate in PBS solution, thereby providing a means to control the lifetime. Long-term cytotoxicity and immune monitoring tests on as-grown monolayer MoS_2_, on ultrasonically dispersed MoS_2_ flakes, and on MoS_2_ dissolved in PBS solutions provide a complete set of information relevant to use in implantable systems. To demonstrate some possibilities in high-performance devices, we fabricated MoS_2_-based bioabsorbable, multifunctional sensors with capabilities for measuring pressure, temperature, strain, and acceleration. Experiments in live animal models illustrate the function of such sensors deployed for measuring temperature in the intracranial space in mice. The results suggest that introduction of 2D semiconducting materials into transient electronic systems may lead to significant opportunities in the development of flexible, stretchable, conformal, and transparent devices and systems with minimal materials load on the environment and/or surrounding biology. Such transient devices may find roles in temporary biomedical implants and resorbable environmental monitors.

## Results

### Dissolution characteristics and behavior of monolayer MoS_2_

To reveal the chemistry and chemical kinetics of dissolution of monolayer MoS_2_ in PBS solution, we first investigated isolated crystals with a small number of grain boundaries (GBs). The combination of highly crystalline structures and well-delineated grains are beneficial for the analyses presented subsequently. Monolayer MoS_2_ crystals with sharp triangular corners and grain size of 5–25 μm were synthesized on SiO_2_/Si substrates using atmospheric pressure chemical vapor deposition (APCVD) (Supplementary Fig. 1a), as described in the Methods Section. The characteristic Raman peaks are at 382 cm^−1^ (in-plane *E*_2g_ mode) and 402 cm^−1^ (out-of-plane *A*_1g_ mode) (Supplementary Fig. 1b). Such a small difference in frequency (20 cm^−1^) between the two Raman peaks is consistent with the monolayer nature of the crystals^30^. The photoluminescence (PL) spectra support this conclusion, as a sharp excitonic *A*_1_ peak appears at 1.82 eV, indicating a high degree of crystallinity (Supplementary Fig. 1c)^31^. Because the SiO_2_/Si substrate is chemically unstable in PBS solution^8^, the dissolution tests used MoS_2_ crystals transferred onto a sapphire substrate coated with a thin layer of a photodefinable epoxy (SU-8, Microchem Corp.) with smooth surface (root mean square (RMS) roughness ~0.258 nm) to enable high-resolution atomic force microscopy (AFM).

**Fig. 1:**
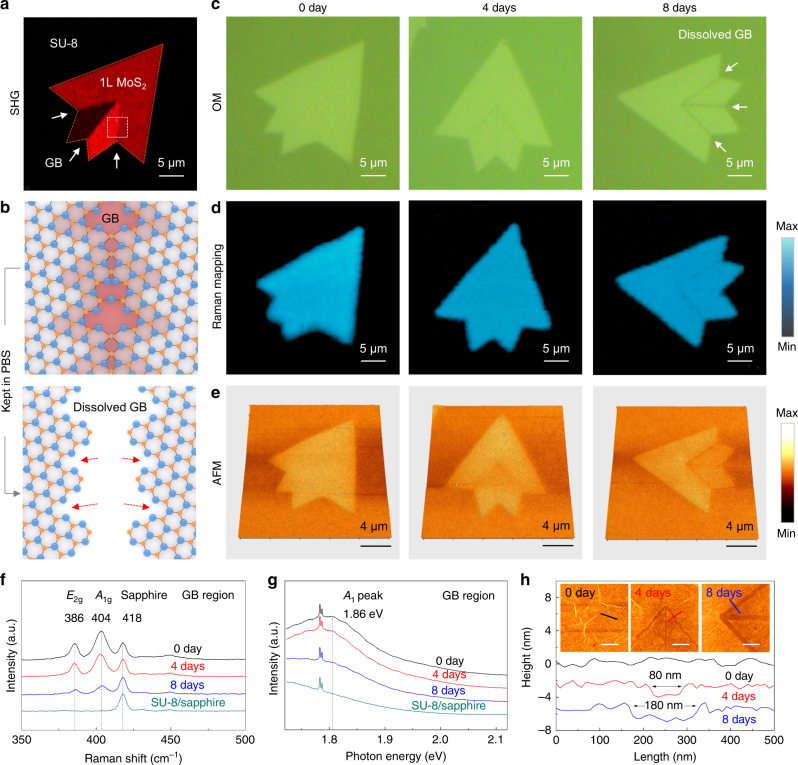
Morphological and structural evolution of isolated monolayer MoS_2_ crystals with different dissolution times in PBS solution. **a** SHG image of an APCVD-grown polycrystalline monolayer MoS_2_ crystals on an SU-8/sapphire substrate, showing the distribution of GB regions. **b** Schematic illustration of the MoS_2_ GB marked in the dotted box of (**a**) before and after dissolution in PBS solution. **c**–**e** OM (**c**), Raman *A*_1g_ intensity mapping (**d**), and AFM (**e**) images of monolayer MoS_2_ crystals, immersed in a PBS solution (pH 7.4) at 75 °C with different dissolution times (0–8 days). **f**–**h** Time-dependent Raman spectra (**f**), PL spectra (**g**), and AFM height profiles (**h**) of the dissolved GB regions. Insets of **h** are the magnified AFM images of the GB regions from **e** (scale bar is 500 nm), and three solid color lines are the scanning places for the corresponding AFM height profiles

The dissolution tests involved monolayer MoS_2_ crystals with the shapes of arrowheads. Second harmonic generation (SHG) nonlinear optical analysis reveals MoS_2_ grains with different orientations and the distribution of GBs^32^, as shown in Fig. 1a. Differences in SH intensity highlight three constituent domains, with GBs in between. Regions of lattice mismatch, defects and dislocations appear at the GBs, including S single (V_S1_) and double vacancies (V_S2_), and Mo dangling bonds (Fig. 1b)^33,34^. For the defect-free areas, the kinetic energy barrier is 1.59 eV, and the chemical stability is high at ambient conditions. A V_S1_ or V_S2_ defect on the MoS_2_ surface reduces the kinetic energy barrier to <0.8 eV, thereby creating a local area with increased reactivity, commonly passivated with oxygen^35,36^. Upon immersion in PBS solution, the defect-rich GBs have increased rates of reaction with water and ions compared to pristine surfaces.

These inferences are supported by accelerated dissolution tests of MoS_2_ crystals in PBS solution (pH 7.4) at 75 °C for 8 days, as determined by morphological and structural measurements every 4 days. Figure 1c–e shows optical micrographs (OM), Raman *A*_1g_ intensity maps and AFM images of MoS_2_ crystals throughout this period. The results indicate clearly that the GB regions dissolve first and serve as the starting points of chemical reactions that extend slowly toward the crystalline regions (Fig. 1c–e, Supplementary Fig. 2). Moreover, continuous reductions in the intensities of the Raman and PL peaks in the GB regions are consistent with this overall conclusion (Fig. 1f–g). The magnified AFM images provide further support (Fig. 1h). Specifically, after 4 days, a gap with a width of ~80 nm appeared, which widened to ~180 nm after 8 days.

As is well known, MoS_2_ monolayer has two phases: trigonal prismatic (labeled as 2H) and octahedral (labeled as 1T). The 2H-MoS_2_ is relatively stable, but semiconductor with poor conductivity. The 1T-MoS_2_ is metastable at room temperature, but metallic material with good conductivity^7^. Given the bandgap at 1.81 eV in Fig. 1g, we can infer that the CVD-grown monolayer MoS_2_ crystals are in 2H phase with semiconducting property^33^. Due to the electronic structure of 2H-MoS_2_, the chemical bonds between Mo and S atoms are robust in an aqueous medium but can be broken by the presence of strong reducing agents, such as alkali metals (Li, Na or K)^37–39^. The existence of Na ions leads to lattice distortions of MoS_2_ and formation of Na_2_S via conversion of pristine 2H-MoS_2_ to 1T-NaMoS_2_ and finally to Na_2_S (see the reaction equation 1 and 2 in Supplementary Note 1)^39,40^. Therefore, an increase in the concentration of Na and K ions will accelerate the degradation processes. Moreover, MoS_2_ will be oxidized by the O_2_ in the PBS solution to yield MoO_4_^2−^, which itself can readily dissolve (see the reaction equation 3 in Supplementary Note 1). Based on the empirical kinetic equation 4 in Supplementary Note 1, the increment in pH value caused by an increase in the concentration of OH^−^ ions can further accelerate the reaction rate^28^.

Because of these combined chemical effects, after immersion for 21–35 days, the dissolved GB regions widen to 1–2 μm and localized holes begin to appear in the central areas of the crystals, likely due to reactions that initiate at point defects (V_S1_ or V_S2_) (Supplementary Fig. 3a–b). Complete dissolution occurs within 49 days (~0.016 nm day^−1^) (Supplementary Fig. 3c). The overall conclusion is that dissolution of monolayer MoS_2_ crystals in PBS occurs as a defect-induced etching process^41^. Due to the reduced kinetic energy barrier, the MoS_2_ GBs dissolve first, followed by point defects, and then regions that extend along the horizontal direction perpendicular to the broken edges, eventually merging and leading to complete dissolution. The rate of this dissolution across a single crystalline domain of MoS_2_ (from edge to center) is 10–20 nm day^−1^ in PBS solution (pH 7.4) at 75 °C. For polycrystalline monolayer MoS_2_, the above result suggests that the total dissolution time can be decreased with smaller grain size, thereby providing an approach for tuning the lifetime of bioabsorbable electronic devices. For this reason, investigations reported here focus on large-scale, continuous MoS_2_ films with polycrystalline texture and small grain size.

Low-pressure chemical vapor deposition (LPCVD) provides a scalable means for synthesizing such films (for more information on this process, please refer to the Methods Section). With a seed layer, large-scale, continuous films of MoS_2_ can be grown on SiO_2_/Si substrates (Supplementary Fig. 4a). However, such MoS_2_ films are typically polycrystalline, thus GBs are easily formed because of the merger of crystalline domains with different orientations. The GBs significantly affect the mechanical, optical, thermal, and electrical properties due to the associated defect distribution^34^. For instance, monolayer MoS_2_ with smaller grain size exhibits lower mobility, resulting from the extra scattering for electron transport but more uniform electrical properties^23^. Thus, it is feasible to tailor the properties of 2D MoS_2_ and its devices via defect engineering. We studied the large-scale MoS_2_ continuous film with an average grain size of ~200 nm, which possesses proper properties for target devices (Supplementary Fig. 4b). Compared with the isolated crystals, Raman spectrum of such continuous film also shows two characteristic peaks corresponding to *E*_2g_ and *A*_1g_ modes, and the frequency difference between these two modes is ~20 cm^−1^ (Supplementary Fig. 4c), indicating that the film is monolayer indeed^24^. Besides, the film demonstrates a sharp *A*_1_ peak at 1.87 eV (Supplementary Fig. 4d), suggesting a relatively high quality but small grain size that are promising for the bioabsorbable application^26^. X-ray photoemission spectroscopy (XPS) spectra show an S2s peak at 226.7 eV and Mo 3d peaks at 229.5 and 232.6 eV, corresponding to the 3d_5/2_ and 3d_3/2_ doublets, respectively (Supplementary Fig. 4e). Peaks at 162.4 and 163.6 eV arise from the S 2p_3/2_ and 2p_1/2_ orbitals, respectively (Supplementary Fig. 4f)^24^. The stoichiometric ratio of Mo to S estimated from the respective integrated peak areas of the XPS spectra is 1:1.95, consistent with the presence of the S vacancies in the film^34^.

**Fig. 2:**
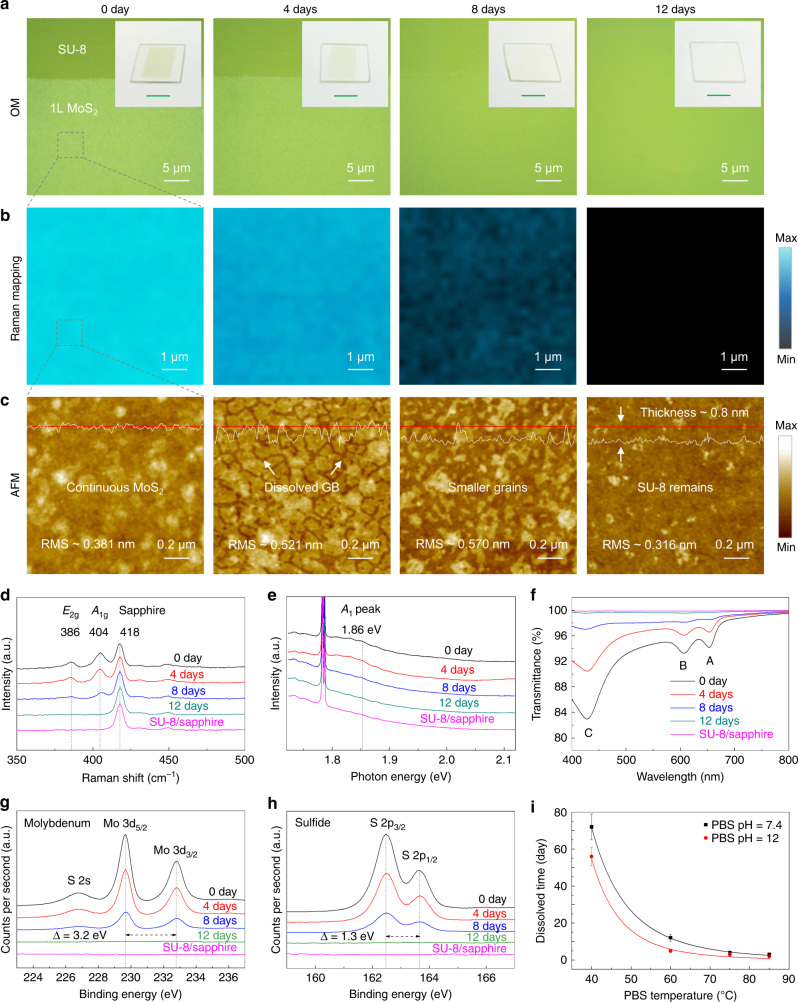
Morphological and structural evolution of continuous monolayer MoS_2_ films with different dissolution times in PBS solution. **a**–**c** OM (**a**), Raman *A*_1g_ intensity mapping (**b**), and AFM (**c**) images of LPCVD-grown polycrystalline monolayer MoS_2_ (grain size ~200 nm) immersed in a PBS solution (pH 7.4) at 60 °C with different dissolution times (0–12 days). Insets are the corresponding optical images of the dissolved MoS_2_ monolayers on SU-8/sapphire substrates (scale bar is 5 mm). **d**–**h** Time-dependent Raman (**d**), PL (**e**), transmittance (**f**), and X-ray photoemission spectroscopy (**g**,** h**) spectra of the dissolved MoS_2_ monolayers. **i** Temperature-dependent and pH-dependent speed at which the MoS_2_ monolayer dissolved in PBS solution. Error bars of the experimental data represent standard error of mean

Figure 2a–h shows the results of studies of dissolution of MoS_2_ monolayer films in PBS solution (pH 7.4) at 60 °C for 0–12 days. These figures include images obtained through OM, Raman *A*_1g_ intensity mapping, AFM and the related Raman, PL, transmittance, and XPS spectra collected every 4 days. As time passes, the continuity of the MoS_2_ film decreases gradually and then vanishes after 12 days (~0.067 nm day^−1^), which is four times faster than the one of the isolated crystals (Fig. 2a). The continuous loss of Mo and S atoms during the dissolution process leads to expected changes in the intensity maps of the Raman *A*_1g_ peak (Fig. 2b). The AFM images in Fig. 2c show that the film dissolves in three steps: first, the GBs dissolve, thereby isolating the grains; second, the edges of the grains continue to react, leading to a reduction in their sizes; and third, the MoS_2_ grains dissolve entirely. It is obvious that the total dissolution time of polycrystalline monolayer MoS_2_ in PBS solution is fundamentally determined by its grain size. Smaller grains need a shorter time to be etched from the edge to the center with the rate of 10-20 nm day^−1^. Besides, the surface roughness increases from 0.36 to 0.57 nm after 8 days, due to steps and edges that appear near the GBs. After 12 days, the RMS roughness rapidly decreases to 0.32 nm, comparable to that of the underlying layer of SU-8 (0.26 nm), suggesting full dissolution (Supplementary Fig. 5). The Raman and PL spectra are consistent with these observations; characteristic peaks for MoS_2_ disappear after 12 days (Fig. 2d–e). It should be noted that there is not obvious Mo-O related Raman peak located between 200–300 cm^−1^ (Supplementary Fig. 6a).

As the area of exposed SU-8 increases, the light transmittance and absorption increase and decrease, respectively (Fig. 2f). The concentration of Mo in PBS solution before and after monolayer MoS_2_ dissolution was measured by using inductively coupled plasma mass spectrometry (ICP-MS) (Supplementary Fig. 6b). Before the dissolution, Mo concentration in original PBS solution was 2.736 ppb. After one and five pieces of MoS_2_ monolayer samples (1 × 1 cm) dissolved in PBS solutions, the related Mo concentrations increased from 4.919 to 14.303 ppb (5 times), which means that the Mo atoms transfer from the SU-8 substrate to the PBS solution. Besides, the continued dissolution of the MoS_2_ samples increases the Mo concentration. We further evaluated the elemental composition and chemical state of the dissolved MoS_2_ film via XPS (Fig. 2g–h). The results show that the peak positions remain constant and their amplitudes decrease, indicating no changes in either the bandgap or the doping level or type^42^. Besides, Mo-O bonding peaks were also not observed at ~233 and ~236 eV in the XPS spectra, regardless of the dissolution time, because the intermediate, ionic molybdate tetroxide created from the oxidation of Mo spreads quickly into the PBS solution^28^. Other relevant peaks, such as P 2p (133.3 eV) and Na 1s (1071.7 eV), increase slightly, as a possible consequence of solute adsorption (Supplementary Fig. 6c-e). In other words, the dissolution products remain in the solution rather than on the substrate.

A series of dissolution tests performed in PBS solutions at different temperatures and pH levels reveal the effects of these parameters on the rates (Fig. 2i). Optical images of a sample of MoS_2_ under such different conditions appear in Supplementary Fig. 7. At pH 7.4, complete dissolution requires 3, 4, 12, and 72 days at temperatures of 85, 75, 60, and 40 °C, respectively. As the pH increases to 12 through the addition of NaOH solution (0.1 mol L^−1^), the dissolution time decreases to 2, 3, 5, and 56 days under the same temperatures. At 40 °C, the dissolution rates at these two pH levels are ~0.011 and ~0.014 nm day^−1^, which are much slower than that of Si NMs (ranging from a few nanometers to hundreds of nanometers day^−1^)^14^. Note that the degradation process slows as the temperature of PBS solution (pH 7.4) reduces to 37 °C (~99 days) or even 30 °C (~200 days), as expected in Supplementary Fig. 8. The activation energies (~105 meV at pH 7.4 and ~147 meV at pH 12) are extracted by fitting the temperature-dependent dissolving rate as shown in the Fig. 2i.^14^ Additional details about the fitting are provided in Supplementary Fig. 9. It should be noted that the addition of NaOH solution increases the concentration of both Na^+^ and OH^−^ ions. Therefore, for this system, the observed acceleration in dissolution with increasing pH results from effects of both Na^+^ and OH^−^, per the reaction equations 2 and 4 as shown in Supplementary Note 1.

### Cytotoxicity and immune monitoring tests of monolayer MoS_2_

The biocompatibility of the starting materials and the products of dissolution are critically important for applications in implantable electronics. Recent studies report results on the toxicity of synthetic MoS_2_ using assays based on salivary gland cells, A549, and HEK293f cells^28,43–45^. Here, we explore the cytotoxicity of MoS_2_ both before and after their dissolution, using starting materials as continuous films, isolated flakes, and as pre-dissolved in PBS solution.

**Fig. 3:**
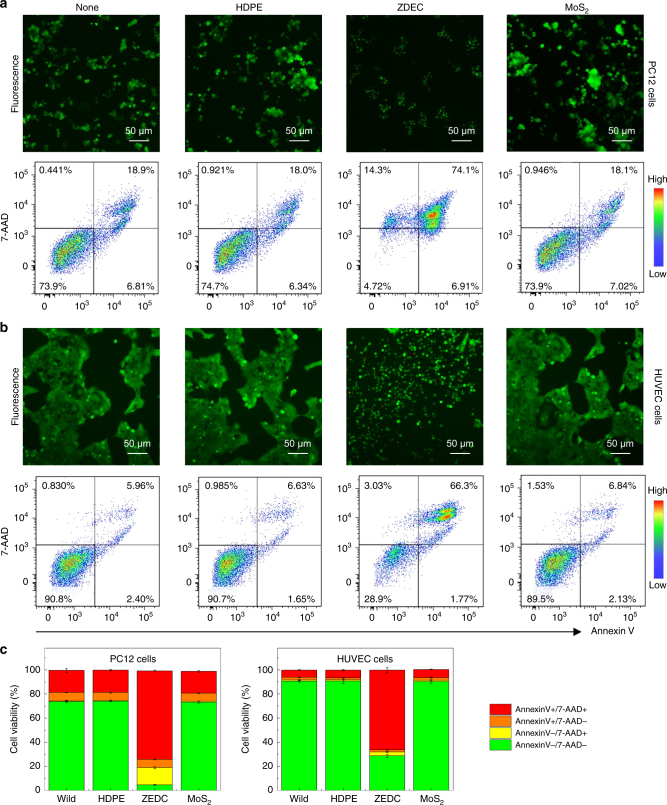
In vitro biocompatibility tests of the polycrystalline MoS_2_ monolayer. **a**,** b** Fluorescence images (top) and apoptotic/necrotic cell death profiles analyzed by flow cytometry (bottom) of PC12 pheochromocytoma neuronal origin cells (**a**) and HUVEC endothelial cells (**b**) upon 24 h culture with none, HDPE, ZDEC, and MoS_2_ continuous films. **c** Cell viability assessed as AnnexinV−/7-AAD− live, AnnexinV+/7-AAD− early apoptotic, AnnexinV+/7-AAD+ late apoptotic, and AnnexinV−/7-AAD+ necrotic cell populations in each condition is averaged and plotted as bar graphs with standard error

Figure 3a shows fluorescence images (top) and apoptotic/necrotic cell death profiles analyzed by flow cytometry (bottom) of PC12 pheochromocytoma neuronal origin cells upon 24 h culture with none, HDPE, ZDEC, and MoS_2_ continuous films. While PC12 cells cultured on HDPE and MoS_2_ film did not show any detectible changes in morphology (top) or AnnexinV/7-AAD (7-Amino-Actinomycin D) staining (AnnexinV−/7-AAD− live, AnnexinV+/7-AAD− early apoptotic, AnnexinV+/7-AAD+ late apoptotic, and AnnexinV−/7-AAD+ necrotic cell populations), those cells cultured on ZDEC demonstrated less than 5% viable cells (AnnexinV−/7-AAD− live population). Similarly, HUVEC cells cultured on ZDEC film showed significantly high percentages of dying cells (AnnexinV+/7-AAD+ late apoptotic), while those on HDPE or MoS_2_ did not show any changes in viability (Fig. 3b). The results from three independent experiments were plotted as bar graphs in Fig. 3c.

**Fig. 4:**
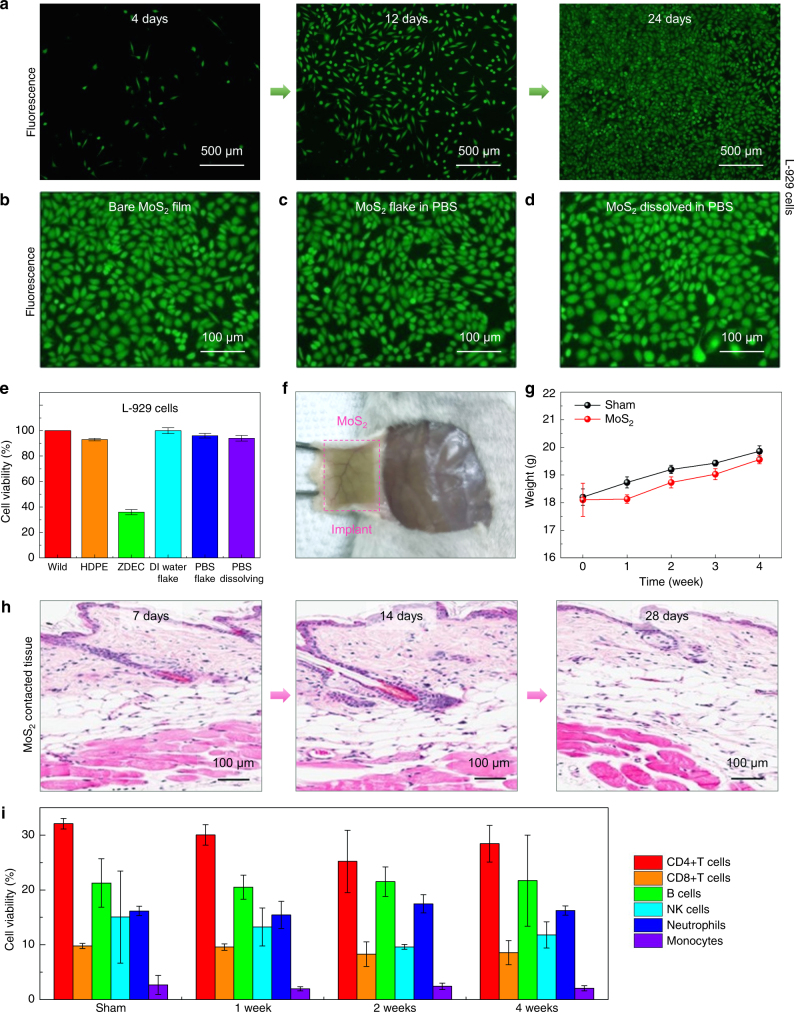
In vitro and in vivo long-term biocompatibility tests of the polycrystalline MoS_2_ monolayer. **a** Fluorescence images of cells on a MoS_2_ monolayer following in vitro culture for 4, 12, and 24 days. **b**–**d** Fluorescence images of cells following 24 days of culture with different MoS_2_ states, including MoS_2_ continuous film (**b**), PBS with MoS_2_ flakes (**c**), and dissolved MoS_2_ (**d**). **e** Cell viability of L-929 cells, cultured for 24 h in media containing HDPE or ZDEC, MoS_2_ flakes in DI water, MoS_2_ flakes in PBS, and MoS_2_ dissolved in PBS, was assessed by the CCK-8 assay. **f** Images of MoS_2_ film implanted in the subdermal dorsal region of BALB/c mice for 4 weeks. **g** The body weight of mice after the subcutaneous implantation of devices for 4 weeks. **h** Immunohistochemistry of tissues surrounding the implanted MoS_2_. The individual tissue sections surrounding the implanted MoS_2_ were subjected to H&E staining and photographed. **i** Immunoprofiling of cell populations in peripheral blood after the implantation of MoS_2_ in mice for 4 weeks. The individual immune subsets (CD4, CD8, B cell, NK cell, neutrophil, and monocytes) are presented as a percentage of immune cells found in the peripheral blood. Error bars of the experimental data represent standard error of mean

We next examined the long-term biocompatibility of MoS_2_ films in vitro. To this end, we chose L-929 cells, mouse fibroblast cells, as they are suitable for short-term and long-term tissue biocompatibility tests. Cell growth and proliferation cultured on MoS_2_ atomic layers were analyzed by fluorescence imaging (Fig. 4a). Cells adhere tightly to the MoS_2_ and spread well during the culture period. After 24 days, most cells remain viable and occasionally form clusters, consistent with no adverse effect on cell proliferation in vitro (Supplementary Fig. 10). Analysis of cell growth in culture media with various concentrations of MoS_2_ flakes and end products of MoS_2_ dissolution in PBS solution yield additional insights (Fig. 4b-d, Supplementary Fig. 11). Fluorescence images show no obvious changes in terms of cell growth for both the MoS_2_ flakes and solution with dissolved MoS_2_, as in the results of negative control samples (e.g., HDPE). Cells exposed to PU-ZDEC, however, show damaged cell morphology, as expected for the positive control (Supplementary Fig. 12 and 13)^46^. Staining cells with Annexin V and 7-AAD at 4, 12, and 24 days following their culture on a MoS_2_ layer, reveal that the percentage of live (Annexin V−7-AAD−), early apoptotic (Annexin V+7-AAD−) and late apoptotic (Annexin V+7-AAD+) cells are comparable to those cultured without MoS_2_ (Supplementary Fig. 14). Furthermore, a cell counting Kit-8 (CCK-8) assay, as summarized in Fig. 4e, shows no adverse effects on the cell viability, similar to the finding for HDPE. By contrast, considerably lower cell viability (~36%) was obtained with cells exposed to PU-ZDEC, as expected for this positive control for toxicity.

Implanting samples with MoS_2_ layers on PET substrates subcutaneously into BALB/c mice and monitoring them for four weeks in a specific pathogen-free facility defines the long-term cytotoxicity and biocompatibility in a vital animal model. Based on the rate of dissolution summarized in Fig. 2, the implanted MoS_2_ will gradually dissolve into the interstitial fluid of mice after 4 weeks (Fig. 4f). The body weights of all MoS_2_-implanted mice increase in parallel to those of the sham control groups (Fig. 4g). Figure 4h and Supplementary Fig. 15 show results of immunohistochemical staining of tissues with MoS_2_ implants. No obvious tissue damage appears following implantation up to 28 days, indicating that MoS_2_ does not cause any serious immunologic or inflammatory reactions^46^. Furthermore, lymphocyte distributions in the peripheral blood of MoS_2_ implanted mice are comparable to those in sham control groups (Fig. 4i); the percentages of CD4+T, CD8+T, B, and NK cells, which could potentially initiate immune responses against implanted MoS_2,_ remain unchanged throughout four weeks of implantation. These results demonstrate the safety, biocompatibility, and non-toxicity of transient MoS_2_ bioelectronics, suggesting their suitability for long-term use in the human body.

### Bioabsorbable and multifunctional sensors based on monolayer MoS_2_

**Fig. 5:**
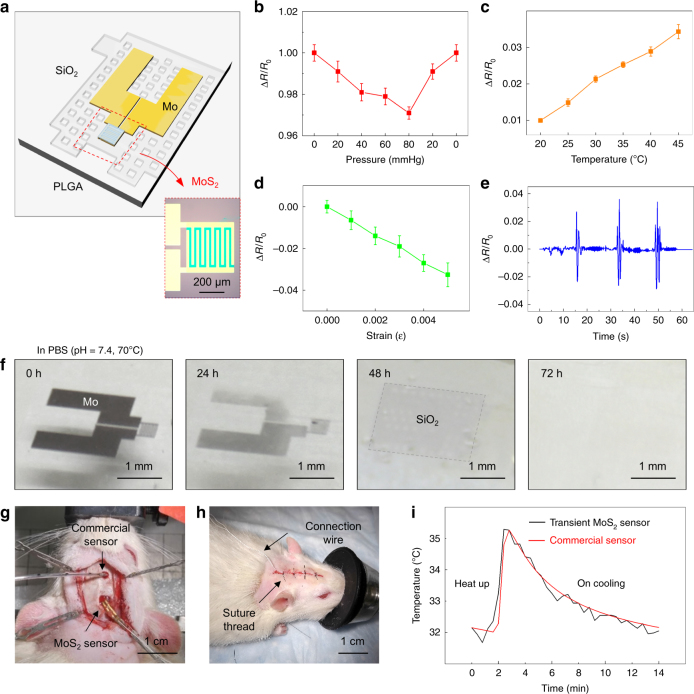
Various applications of MoS_2_-based bioabsorbable sensors. **a** Schematic of the structure of a MoS_2_-based biodegradable sensor. Inset is the OM images of the sensor. **b**–**e** Characteristic responses of the sensor as a function of pressure (**b**), temperature (**c**), strain (**d**), and acceleration (**e**). Error bars of the experimental data represent standard error of mean. **f** Optical images of the sensor in PBS solution (pH 7.4) with different dissolution times (from 0 to 72 h). **g**, **h** Optical images of a MoS_2_-based bioabsorbable sensor implanted in a rat together with a commercial one before (**g**) and after (**h**) suture. **i** In vivo investigation of temperature changes in the intracranial space of the rat model using the implanted sensor, with comparisons to a commercial, non-resorbable device

These findings on the biocompatible and the bioabsorbable nature of MoS_2_ allow its use in implantable, temporary biomedical sensors^7,21,47^. Here, we describe a set of MoS_2_-based bioabsorbable sensors capable of precision measurements of pressure, temperature, strain, and motion. One area of application is the monitoring of pressure or temperature in the intracranial space during healing from a traumatic brain injury, where the sensor functionality is only necessary during the critical risk period for the patient, typically on a timescale of several days. Figure 5a presents an enlarged schematic illustration and an optical image of a representative device. The construction involves purely bioabsorbable materials, including a PLGA membrane (30 μm thick) as a supporting layer on a substrate of nanoporous silicon, with Mo as the electrode. The top and bottom layers of SiO_2_ (about 100 nm thick)^15,48^ provide electrical passivation and act as barriers against biofluids. Details on the materials and fabrication strategies appear in the Methods Section.

To yield a mechanism that is responsive to the pressure of a surrounding biofluid, an etched structure of relief on the surface of the substrate (nanoporous silicon, about 60–80 μm thick) forms a square-shaped air cavity with a depth of 50–60 μm after capping with a film of PLGA. The total size of the substrate is 1.5 mm × 2.5 mm × 0.08 mm and the trench is 0.67 mm × 0.8 mm × 0.03 mm. A layer of MoS_2_ with an interdigitated geometry acts as a piezoresistive sensor near one of the edges of the cavity, where the bending induced strains are maximized. The resistance of this sensor decreases linearly across the full range of pressures related to those in the intracranial space (0–80 mmHg). The slope of the response is 65 kΩ (mm Hg)^−1^, consistent with the calculated results^49^, and a gauge factor of ~60 lies within a range of expected values for MoS_2_^21^. Motion sensors can be constructed with this same platform by addition of a test mass of PLGA (i.e., a single-axis accelerometer, Fig. 5b-d). Furthermore, the device exhibits a temperature-dependent resistance that is linear (Fig. 5c), thereby enabling its use as a temperature sensor with a sensitivity of ~51 kΩ/°C. The acceleration sensor can also detect the motion of the sensor with a cantilevered mass of PLGA (30 μm thick) (Fig. 5e).

In addition to the individual constituent materials, the biocompatibility of the entire system is important to consider^49^. The layers of SiO_2_ and Mo and the nanoporous silicon substrate dissolve in PBS solution (37 °C, pH 7.4) at rates of 23, 8, and 10 μm day^−1^, respectively. PLGA (75 to 25, lactide–glycolide) is known to dissolve in biofluids within 4–5 weeks^50^. Figure 5f and Supplementary Fig. 16 show a series of optical images of a typical device at various times after immersion in a PBS solution at 70 °C. A bioabsorbable polymer (polyanhydride) serves as an encapsulation layer. After dissolution and water penetration through this layer after a few days, the Mo components dissolve (within 48 h), followed by the SiO_2_ layers (within 72 h). Based on results described in previous sections, the MoS_2_ monolayer will completely dissolve within several months, which is somewhat longer than those for the PLGA^51^. Therefore, after the metal electrode and PLGA disappear, the MoS_2_ remains but likely mechanically fractures into small flakes that will dissolve over ~2–3 months. The longevity of the monolayer MoS_2_ in biofluids facilitates the construction of bioabsorbable electronics and biosensors with longer lifetimes than those with Si NMs. The ability to tune the dissolution rate through control of the grain sizes and the defect densities represents an attractive feature in this context.

Sensors implanted into the intracranial space of animal models (mice) allow demonstrations of modes of use that are relevant for monitoring of patients during a recovery period following a severe traumatic brain injury. Measurements of temperature in this context provide an example. Figure 5g–h outlines the surgical process for implantation. Dissolvable surgical glue prevents leakage of cerebrospinal fluid from the intracranial region. Figure 5i summarizes measurements of temperature using this platform, along with comparisons to data obtained with a commercial, non-resorbable device. The intracranial temperature was varied and controlled via heating or cooling blanket placed under the animal. These results show that real-time in vivo monitoring of biological signals by an entirely bioabsorbable sensor correlate well with results from a conventional device.

## Discussion

In summary, the studies described here yield a broad collection of results related to materials, device, and applications aspects of the use of MoS_2_ as isolated crystals and large-area polycrystalline films in biodegradable electronic systems. The results reveal that dissolution in simulated and actual biofluids begins at the defect-rich areas of the material, such as the GBs and locations of point defects. More than 2 months are needed to completely dissolve a representative polycrystalline MoS_2_ monolayer (grain size ~200 nm) in PBS solution at 37 °C, where the rates depend on temperature, pH and the type, and concentration of ions in the solution. Further results demonstrate that monolayer MoS_2_ is a biocompatible semiconductor, based on analyses of long-term tests in cell level assays, and of in vivo immunological evaluations in live animal models, thereby suggesting suitability for use in bioelectronics. At the device level, implantable biomedical sensors based on CVD-grown monolayer MoS_2_ can monitor intracranial temperature well over a specified period before dissolving completely. Applications in bioabsorbable and biocompatible transient sensors, which are capable of monitoring critical parameters in the intracranial space associated with recovery from traumatic brain injury, demonstrate the type of applications of interest in clinical medicine. These findings pave the way for the integration of 2D materials into transient, water-soluble electronic platforms, and for their use in biologically and environmentally resorbable technologies.

## Methods

### Synthesis of monolayer MoS_2_ isolated crystals and continuous films

In this study, we prepared isolated crystals and continuous films of monolayer MoS_2_ on SiO_2_/Si substrates by using APCVD^22^ and LPCVD methods^23,24^, respectively. Before synthesis, the SiO_2_ (300 nm)/Si substrates were cleaned by ultrasonication in acetone, isopropyl alcohol, and deionized (DI) water for 10 min each. The SiO_2_/Si substrates were then immersed in a Piranha solution at 70 °C for 2 h, and subsequently, perylene-3,4,9,10-tetracarboxylic acid tetrapotassium salt (5 μM aqueous solution, 2D semiconductor) was spin-coated onto their surfaces as a seeding solution. We synthesized monolayer MoS_2_ isolated crystals in a single-zone CVD furnace using the APCVD approach^22^. MoO_3_ powder (10 mg, ≥99.5%, Sigma-Aldrich) was placed in an alumina boat and loaded into the center of the furnace chamber. The treated SiO_2_/Si substrate was covered on the boat in an upside-down manner. Another alumina boat containing S powder (1 g, ≥99.5%, Sigma-Aldrich) was loaded 15 cm away from the center of the furnace. The temperature and deposition time was 750 °C and 30 min, respectively, and the heating rate was 50 °C/min. An Ar:H_2_ (50:7.5 sccm) gas mixture was used as the carrier gas to reduce the atmosphere to promote the reaction. After synthesis, the furnace was cooled naturally to room temperature. We then fabricated monolayer MoS_2_ continuous films in a three-zone CVD furnace using the LPCVD method^23,24^. S (1.4 g) and MoO_3_ (35 mg) powders were placed separately in two small quartz tubes upstream of the furnace, and the SiO_2_/Si substrate was placed downstream while facing upstream. The temperatures used in zone 1 (S powder), zone 2 (MoO_3_ powder), and zone 3 (SiO_2_/Si substrate) were 120, 590, and 700 °C, respectively. During the synthesis process, the horizontal reaction chamber was maintained at a low pressure (0.7 Torr) with 100 sccm Ar as a carrier gas. After 40 min, the furnace was turned off and cooled down slowly to room temperature.

### Dissolution test for monolayer MoS_2_ isolated crystals and continuous films

Note that both SiO_2_ and Si dissolve in PBS solution. Therefore, before starting the dissolution test, we transferred the as-grown monolayer MoS_2_ from SiO_2_/Si to SU-8/sapphire because the latter has much higher stability than the former in PBS. To create the SU-8/sapphire substrate, we cleaned sapphire in the same way as we cleaned SiO_2_/Si, and then spin-coated it with SU-8 (10% at 3500 rpm, MicroChem). We then soft-baked the substrate for 5 min at 65 °C, treated it with UV light for 2 min, and then hard-baked it for 30 min at 150 °C. The final thickness of the SU-8 layer was ~100 nm. A poly(methyl methacrylate) (PMMA) solution was spin-coated onto MoS_2_ as a supporting layer, and the SiO_2_ layer was etched away using a diluted HF solution, followed by rinsing in DI water. The PMMA/MoS_2_ film was transferred to the SU-8 layer and dried naturally (>8 h). After removing the PMMA in acetone, the monolayer MoS_2_ crystals and films were successfully transferred on the SU-8/sapphire substrate with excellent adhesion.

To investigate the dissolution process and behavior of the MoS_2_ monolayer, we performed a series of dissolution tests in 60 mL of 1.0 M PBS (Sigma-Aldrich) on a hot plate with different temperatures, pH levels, and times. The PBS solution was replaced after every 2 days to maintain a same concentration of the solution throughout the test. For monolayer MoS_2_ crystals, an accelerated dissolution test was carried out in a PBS solution (pH 7.4) at 75 °C for 49 days. The dissolution test for the monolayer MoS_2_ continuous films was performed in PBS solutions (pH 7.4–12) at 40–75 °C for 72 days. The pH level was controlled by adding a suitable amount of NaOH solution (0.1 mol L^−1^). At the end of the dissolution tests, the MoS_2_ samples were removed from the PBS solutions, rinsed with DI water, and blow-dried using N_2_ gas.

### Characterization of monolayer MoS_2_ before and after the dissolution tests

The morphology of monolayer MoS_2_ was investigated both before and after the dissolution tests by using an optical microscope (Eclipse LV100ND, Nikon) and an atomic force microscope (NX10, Park system). To reveal and confirm the location of the GB in MoS_2_ crystals, a dual-mode erbium-doped fiber laser (Insight Deepsee Dual, Spectra-Physics) was combined with a confocal microscope (IX83, Olympus) to create SHG nonlinear optical images of the crystals. Real-time SHG images were observed after the two spatially overlapped beams were directed to a galvanometric *x*-*y* directional mirror controlled by a scanning system (Fluoview1000, Olympus). The Raman and PL spectra and related mapping images were observed using a Raman microscopic system that had a laser wavelength of 532 nm and power of 0.4 mW (Uni-RAM2, UniNanoTech). The transmittance spectra of the monolayer MoS_2_ continuous films were measured by a UV–Vis spectrophotometer (V-650, JASCO). The concentration of Mo in PBS solution before and after monolayer MoS_2_ dissolution was measured by using ICP-MS (7900, Agilent). In addition, XPS (K-alpha, Thermo VG) was used to investigate the binding energies of Mo and S in the monolayer MoS_2_ films that were produced using different dissolution times.

### Cell culture and direct contact tests

Mouse fibroblasts from a clone of strain L (NCTC clone 929, KCLB-10001; KCLB, Korea) were cultured in a supplemented Eagle’s minimum essential medium (MEM, 10% fetal bovine serum (FBS), L-glutamate, penicillin, and streptomycin), rat adrenal gland PC-12 (CRL-1721; ATCC, USA) were cultured in a supplemented RPMI-1640 Medium essential medium (RPMI-1640, 10% FBS, L-glutamate, penicillin, and streptomycin), and human endothelial HUVEC (PCS-100-013; ATCC, USA), were cultured in a supplemented F-12K Medium essential medium (F-12K, 10% FBS, L-glutamate, heparin, ECGS, penicillin, streptomycin), and incubated at 37 °C in a humidified 5% CO_2_ atmosphere. Each cell was pre-cultured for 24 h in culture medium, which was supplemented with a 10% FBS in 24-well plates and exposed for 24 h to the samples placed in the center of each well, which meant that they covered 10% of the cell layer surface. The morphological changes indicating cytotoxicity and cell growth characteristics were recorded as photographs by using an inverted microscope (CKX41, Olympus, Tokyo, Japan). A live/dead assay (Invitrogen, Carlsbad, CA) was used to test cell viability after extended on-chip culturing. Continuous monolayer MoS_2_ film with L-929, PC-12, and HUVEC cells were incubated with a 1 μM acetol methoxy derivative of calcein (calcein AM, green; live) and 2 μM ethidium homodimer (red; dead) for 35 min in a PBS solution. The cells were then rinsed twice with PBS before the samples were immediately imaged by the inverted microscope; green fluorescence indicated that the cells were viable, while red fluorescence meant that the cells were dead. The images were used to count and calculate the densities of cells in the fluorescein isothiocyanate (FITC, green; live) and tetramethylrhodamine (TRITC, red; dead) channels. The ratio of the integrated density in the FITC to TRITC channel defined the cell viability. The viability of the L-929 cells that came in contact with the sample materials and devices was measured by CCK-8 (Dojindo Laboratories, Kumamoto, Japan) according to the manufacturer’s instructions. To summarize, L-929 cells were incubated with the HDPE, ZDEC, MoS_2_ flakes in DI water/PBS, and dissolved MoS_2_ in PBS on 24-well plates for 24 h before the addition of 10% CCK-8 solution. Cells were then incubated for an additional 2 h at 37 °C to form water-dissoluble formazan. A 50 μL formazan solution was then collected from each sample and added to one well in a 96-well plate. Three parallel replicates were prepared. The absorbance at 450 nm was determined using a microplate spectrophotometer (iMark, Bio-RAD).

### Flow cytometry with apoptosis assays

Anti-mouse CD3, CD4, CD8, CD19, NK1.1, CD14, Gr-1, and F4/80 monoclonal antibodies (mAbs) conjugated with FITC, phycoerythrin, phycoerythrin-cyanine dye, peridinin chlorophyll protein complex with cyanin-5.5, and allophycocyanin were purchased from eBioscience (San Diego, CA, USA). Isolated cells were resuspended in a 100 μL FAC buffer (PBS containing 2% FBS and 0.02% sodium azide) solution and incubated with anti-CD16/CD32 mAbs (2.4G2, Fc block) to block the FcRIII/II receptors. The cells were then incubated for 20 min at 4 °C. Flow cytometry was performed with FACS Canto II (BD Bioscience, San Diego, CA, USA), and the data were analyzed by the FlowJo software (ThreeStar, USA). Fifty thousand lymphocyte populations gated by forward scatter/side scatter were analyzed. Annexin V-FITC Apoptosis detection kit was purchased from BD Biosciences (San Diego, US). The L-929 cells were then analyzed for their expression of Annexin V and 7-AAD to determine the number of viable cells: Annexin V negative and 7-AAD negative (Annexin V−/7-AAD−); cells undergoing apoptosis, Annexin V positive and 7-AAD negative (Annexin V+/7-AAD−); and dead cells or cells that were in the last stage of apoptosis, Annexin positive and 7-AAD positive (Annexin V+/7-AAD+).

### In vivo tissue biocompatibility tests

Sterile implant samples, each approximately 3 mm × 10 mm, were prepared aseptically. We used 6-week-old female mice, the mouse groups were sham only (*n* = 3), 1 week (*n* = 3), 2 weeks (*n* = 3), and 4 weeks (*n* = 3). The mice were implanted with continuous monolayer MoS_2_ films for four weeks and euthanized prior to the excision of muscle tissues and macroscopic examination of the implant sites. The inserted devices initially adhered to the subcutaneous layers (hypodermis) due to adequate adhesion to the skin layer based on van der Waals forces. A thin transparent layer of fibroblasts was found to have grown over the devices, which helped the inserted devices to stay in place. A microscopic evaluation was then conducted to further determine the tissue response.

### Histological studies

The mice were euthanized via CO_2_ asphyxiation, and the implanted samples and surrounding tissues were excised. The tissues were then fixed in 10% formalin, embedded in paraffin, cut into 4 μm sections, and stained using H&E for histological analysis. The experiments were repeated three times. Statistical significance was determined by one-way analysis of variance followed by Dunnett’s multiple comparison tests. Significance was ascribed at p < 0.05. All analyses were conducted using the Prism software (Graph Pad Prism 5.0).

### Fabrication of MoS_2_-based bioabsorbable sensors

The fabrication began with the spin-coating of polyimide (~1.2 μm, Sigma-Aldrich) and a sacrificial layer of PMMA (~100 nm, MicroChem) on temporary silicon carrier substrates. Electron-beam evaporation was used to develop a layer of SiO_2_ (thickness 100 nm) that could encapsulate the MoS_2_ atomic layer and protect it during reactive ion etching (RIE). Mo was also deposited by electron-beam evaporation for metal electrode. Monolayer MoS_2_ grown by the LPCVD method was transferred and patterned using photolithography and CHF_3_/O_2_ plasma etching (35/15 sccm, 100 W, 10 s). The casting and patterning of a top coating of D-PI (~1.2 μm) facilitated the formation of the MoS_2_ channel and connected it to a place near the neutral mechanical plane. Defining a mesh structure across the multilayer (D-PI/SiO_2_/D-PI/PMMA) by RIE before immersing it in a buffered oxide etchant exposed the base layer of PMMA and allowed it to be released into acetone for transfer onto a PLGA film (~30 μm thick). The whole device was heated to a temperature near the glass transition temperature of PLGA (55–60 °C; the lactide/glycolide ratio was 75:25) to make it more stable. Last, the top layer of the PI was eliminated by RIE.

### Evaluation in animal models

Studies were performed in strict accordance with the recommendations in the “Guide for the Care and Use of Laboratory Animals” of the National Institutes of Health. The protocol was approved by the Institutional Animal Care and Use Committee of Washington University in St. Louis. Male Lewis rats (*n* = 3) weighing 275–300 g (Charles River Laboratories) received subcutaneous injections of buprenorphine hydrochloride (0.05 mg/kg, Reckitt Benckiser Healthcare Ltd, USA) for pain management, and of ampicillin (50 mg/kg; Sage Pharmaceuticals, USA) to prevent infection at the implantation site before the surgical process. Animals were anaesthetized with isoflurane gas (4% for introduction and 2% for maintenance) and held in a stereotaxic frame for the duration of the surgical procedure and measurements. Opening a craniectomy and dural, implanting bioresorbable temperature sensor on the cortical surface, sealing the craniectomy with a biodegradable surgical glue (Histoacrly^R^, Braun Corporation, Spain), and suturing the skin implanted the fully resorbable biosensing device in intracranial space. Comparison testing with commercial thermistor (DigiKey Electronics, USA) implanted in parallel to bioresorbable sensors demonstrated the functionality of the bioresorbable sensors. In vivo functionality tests of temperature sensors involved three trials using different batches of devices and animals for the reproducibility^52,53^.

### Data availability

Data supporting the findings of this study are available within the article and its Supplementary Information file, and from the corresponding authors upon reasonable request.

## Electronic supplementary material

Supplementary Information
