# Supplementary material for: CVD-grown monolayer MoS2 in bioabsorbable electronics and biosensors
Source: Nat Commun. 2018 Apr 27;9:1690. doi: 10.1038/s41467-018-03956-9 (PMC5924366; doi:10.1038/s41467-018-03956-9)
Supplement: Supplementary file 1 — Supplementary Information [file 41467_2018_3956_MOESM1_ESM.pdf]

Supplementary Information

CVD-Grown Monolayer MoS_2 in Bioabsorbable Electronics and Biosensors

Xiang Chen[1†], Yong Ju Park[1†], Minpyo Kang[1†], Seung-Kyun Kang[2], Jahyun Koo[2], Sachin M. Shinde[1], Jiho Shin[2], Seunghyun Jeon[3], Gayoung Park[3], Ying Yan[4], Matthew R. MacEwan[4], Wilson Z. Ray[4], Kyung-Mi Lee[3]*, John A Rogers[2]* & Jong-Hyun Ahn[1]*

[1] School of Electrical and Electronic Engineering, Yonsei University, 50 Yonsei-ro, Seoul 03722, Republic of Korea

[2] Departments of Materials Science and Engineering, Biomedical Engineering, Chemistry, Mechanical Engineering, Electrical Engineering and Computer Science, Center for Bio-Integrated Electronics, Simpson Querrey Institute for Nano/Biotechnology, Northwestern University, Evanston, Illinois 60208, USA.

[3] Department of Biomedical Engineering, College of Health Science, Korea University, Seoul 136-703, Republic of Korea

[4] Department of Neurological Surgery, Washington University School of Medicine, St Louis, Missouri 63110, USA

[†]These authors contributed equally to this work.

*Corresponding authors:

kyunglee@korea.ac.kr, jrogers@northwestern.edu and ahnj@yonsei.ac.kr

Supplementary Notes

Supplementary Note 1. The possible reactions during MoS_2 biodegradation process in PBS solution

Since the dissolution tests of monolayer MoS_2 in this work involved PBS solution, which contains disodium hydrogen phosphate (Na_2HPO_4), sodium chloride (NaCl), potassium chloride (KCl), potassium dihydrogen phosphate (KH_2PO_4), O_2, and H_2O, likely reactions are[1,2]:

$$xM + MoS_2 \rightarrow M_xMoS_2 \quad (1)$$

$$M_xMoS_2 + yM^+ \rightarrow M_2S + Mo \quad (2)$$

$$MoS_2 + 9/2O_2 + 3H_2O \rightarrow MoO_4^{2-} + 2SO_4^{2-} + 6H^+ \quad (3)$$

Where M = Na or K.

Besides, the empirical kinetic equation affected by pH level in PBS solution is[3]:

$$d[MoS_2]/[MoS_2]dt = 1.9*[OH^-]^{0.17},\ 1/d \quad (4)$$

where the units of the dissolution rate and OH^{-1} concentration are $days^{-1}$ and mol/L, respectively.

Supplementary Figures

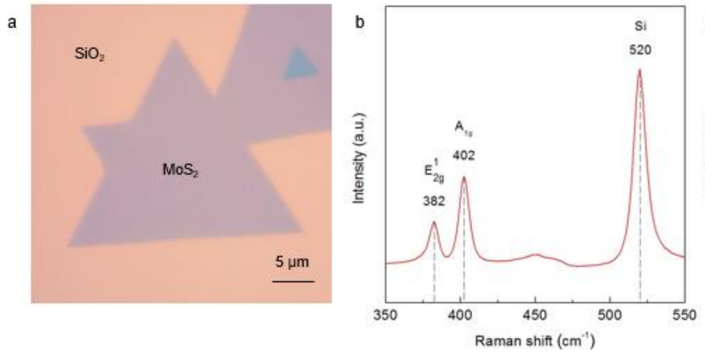

Supplementary Figure 1 | The optical characterization of as-grown monolayer MoS_2 crystals. a, OM image of isolated polycrystalline MoS_2 monolayer fabricated on SiO_2 substrate by using APCVD method; **b**,**c**, Raman (**b**) and PL (**c**) spectra of the corresponding MoS_2 monolayer.

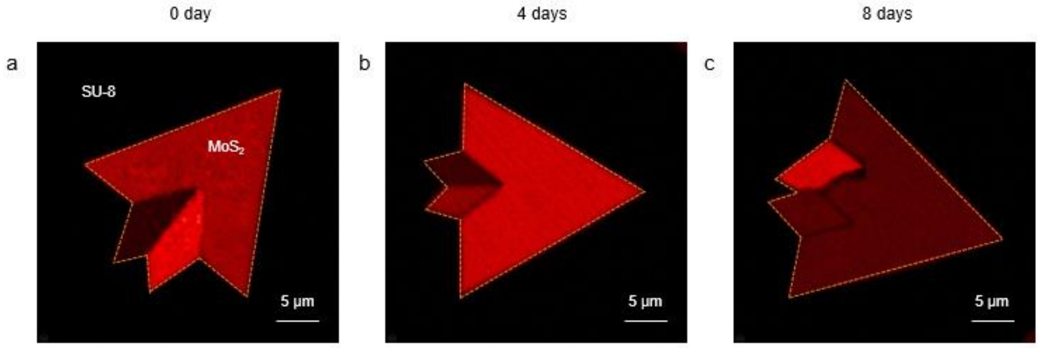

Supplementary Figure 2 | SHG characterization of monolayer MoS_2 crystals with different dissolution time. a-c SHG mapping images of MoS_2 crystals on SU-8/sapphire substrate kept in PBS solution at 75 °C within 0 day (**a**), 4 days (**b**) and 8 days (**c**).

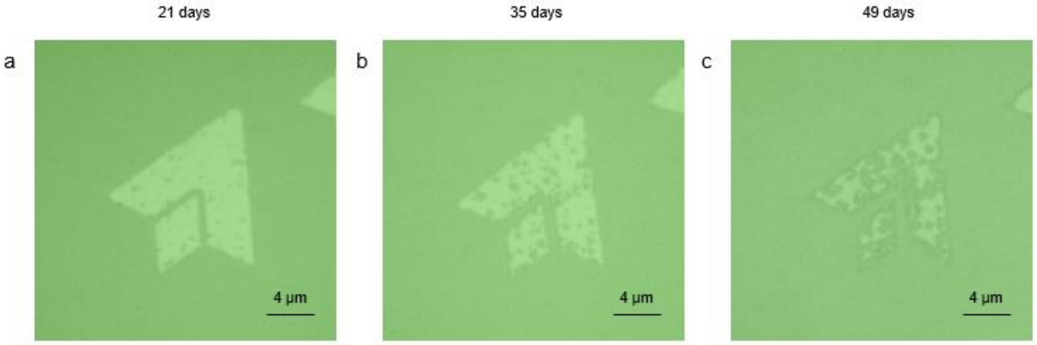

Supplementary Figure 3 | Morphology evolution of monolayer MoS_2 crystals with different dissolution time. a-c OM images of MoS_2 crystals on SU-8/sapphire substrate after kept in PBS solution at 75 °C for 21 (**a**), 35 (**b**) and 49 (**c**) days, respectively.

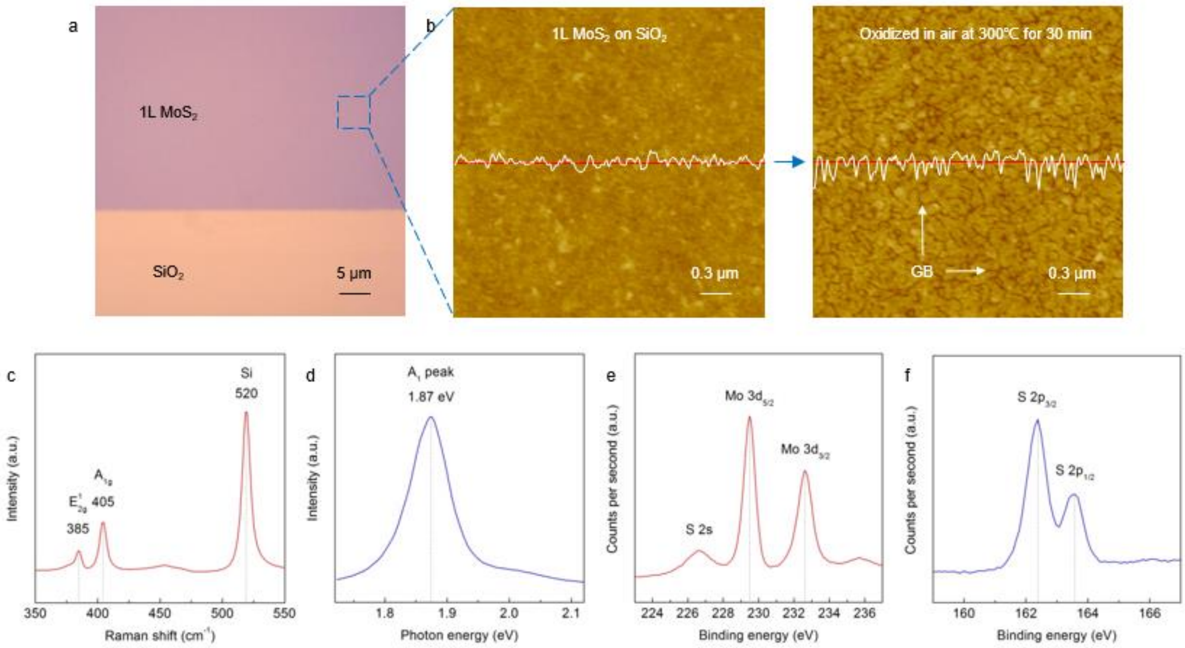

Supplementary Figure 4 | Optical and structural characterization of monolayer MoS_2 continuous film. **a**, OM image of monolayer MoS_2 continuous film on SiO_2 substrate synthesized by using LPCVD method; **b**, AFM images of the related MoS_2 film before and after oxidation to visualize its grain boundary; **c-f** Raman (**c**), PL(**d**) and XPS (**e**,**f**) spectra of the corresponding MoS_2 film.

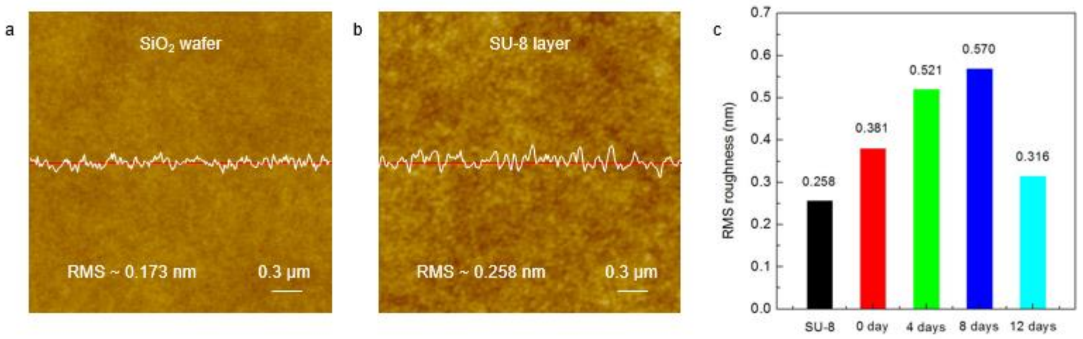

Supplementary Figure 5 | Surface roughness of SU-8 layer and monolayer MoS_2 film with different dissolution time. a,**b**, AFM images of SiO_2 (**a**) and SU-8 (**b**) substrates; **c**, RMS roughness evolution of SU-8 substrate and monolayer MoS_2 on SU-8 with different dissolution times from 0 day to 12 days in PBS solution at 60 ℃.

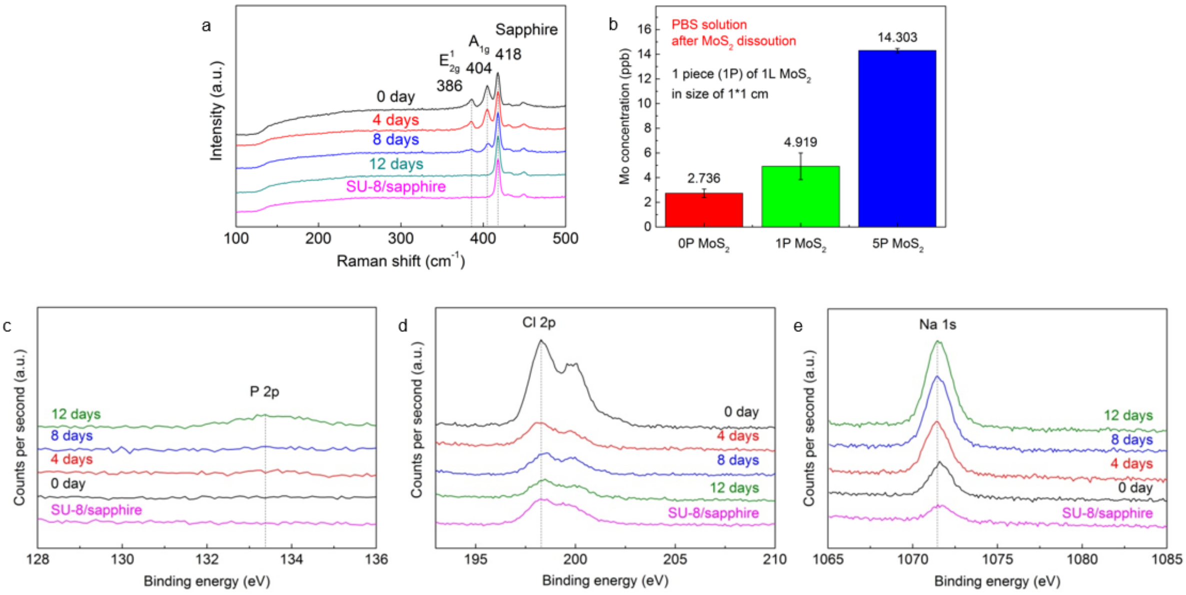

Supplementary Figure 6 | Raman, ICP-MS, and XPS study of monolayer MoS_2 film with different dissolution time or PBS solution. a, Time-dependent Raman spectra of the dissolved MoS_2 monolayers, with Raman shift ranging from 100 to 500 cm^{-1}; **b**, Mo concentration in PBS solution before and after monolayer MoS_2 dissolution, measured by using inductively coupled plasma mass spectrometry (ICP-MS); **c-e**, XPS spectra of P (**c**), Cl (**d**) and Na (**e**) from the monolayer MoS_2 film on SU-8/sapphire with different dissolution time from 0 day to 12 days in PBS solution at 60 ℃.

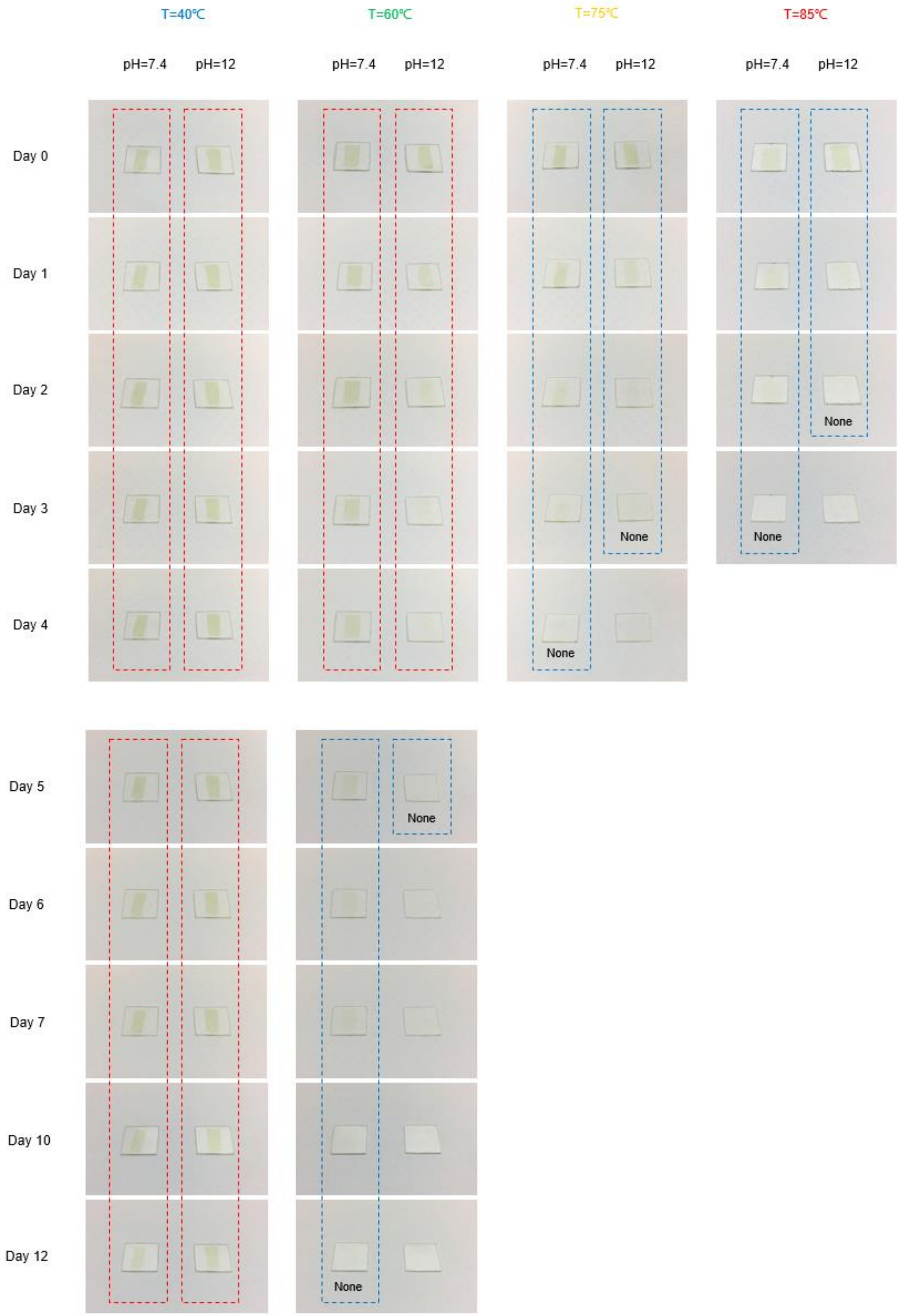
T=40℃
T=60℃
T=75℃
T=85℃
pH=7.4
pH=12
pH=7.4
pH=12
pH=7.4
pH=12
pH=7.4
pH=12
Day 0
Day 1
Day 2
None
Day 3
None
None
Day 4
None
Day 5
None
Day 6
Day 7
Day 10
Day 12
None

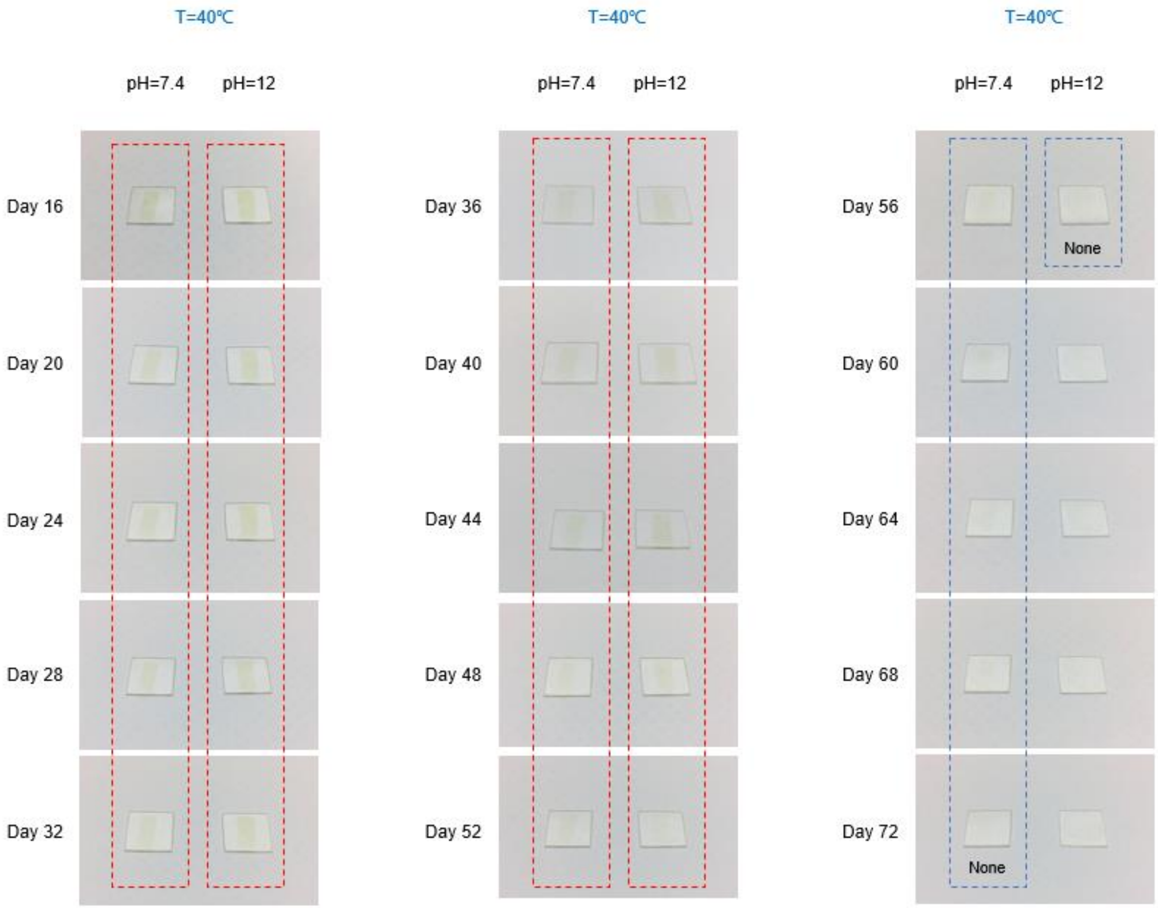

Supplementary Figure 7 | Temperature-, pH- and time-dependent dissolution of monolayer MoS_2 film. Optical images of large-scale MoS_2 monolayers on SU-8/sapphire substrate (1×1 cm) kept in PBS solution with different temperature (40-85 °C), pH level (7.4-12) and time (0-72 days). After 12 days, monolayer MoS_2 films in PBS solution at 60, 75 and 85 ℃ were all degraded except the one at 40 ℃. Since then, we observed the samples at 40 ℃ until the end (72 days).

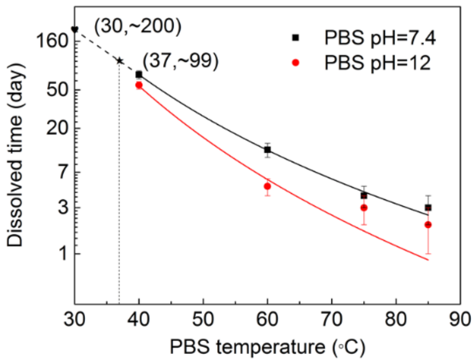

Supplementary Figure 8 | Temperature- and pH-dependent speed at which the MoS_2 monolayer dissolved in PBS solution. The linear scale of y axis has been converted into ln scale. Error bars of the experimental data represent standard error of mean.

$$\ln(r) = (-E_a/K_B) * (1/T) + C$$

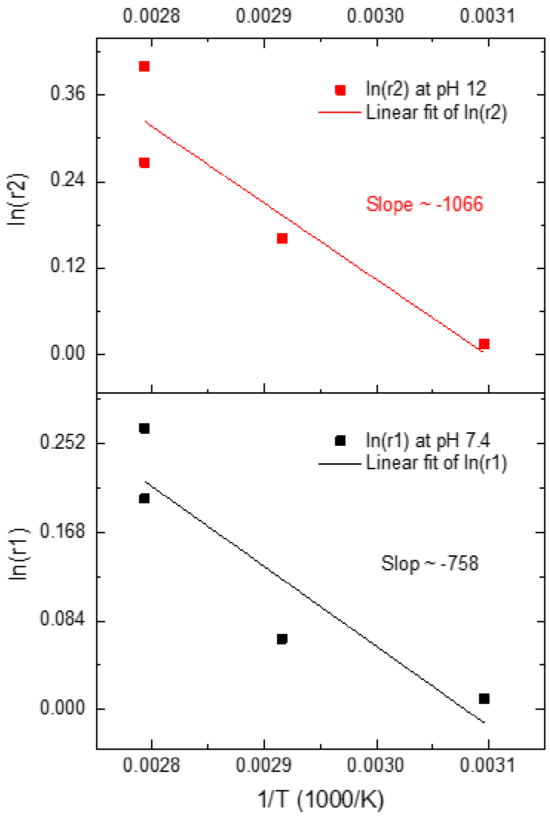

Supplementary Figure 9 | Temperature- and pH-dependent dissolution rate of monolayer MoS_2 film. Plots of the natural logarithm of the monolayer MoS_2 dissolution rates (r) as a function of the inverse of the temperature (T) at pH 7.4 and 12. The slope of the linear fitting estimates the activation energy as shown in the equation (Top of the figure). The equation was derived from the previous reported research (Ref. 14 in main text).

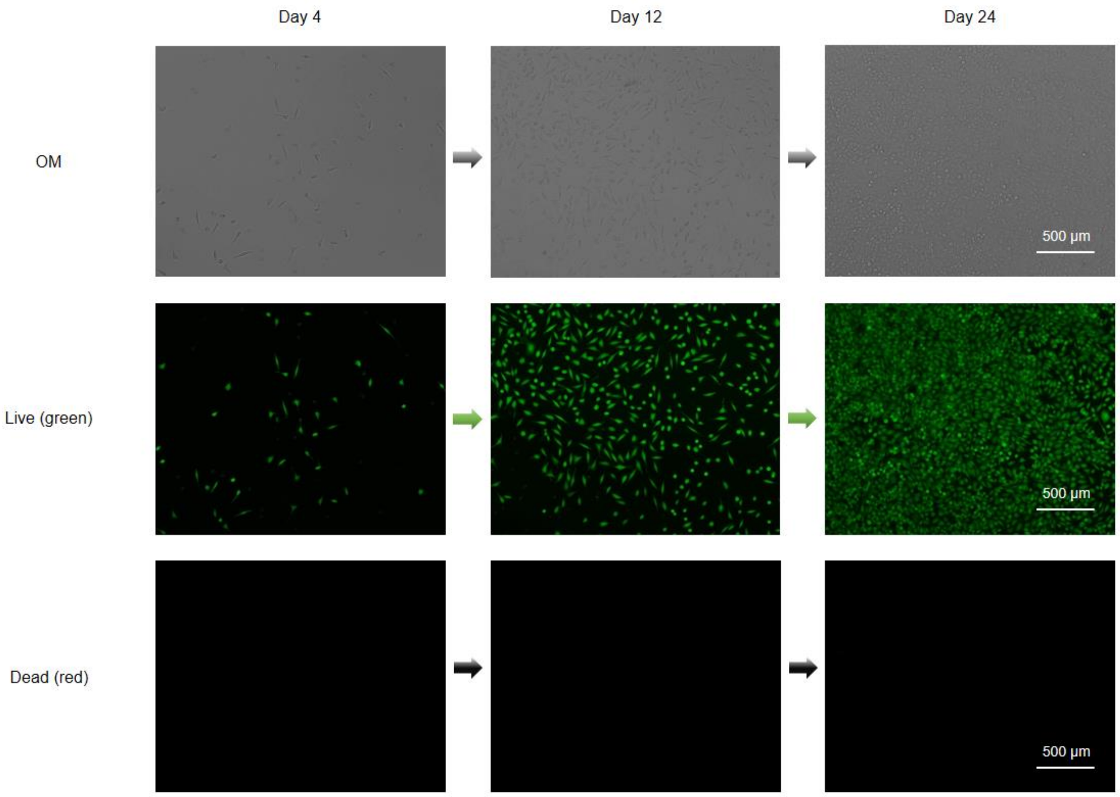

Supplementary Figure 10 | Time-dependent cell culture on monolayer MoS_2 film. OM and fluorescent images of cell culturing on monolayer MoS_2 film for 4, 12 and 24 days.

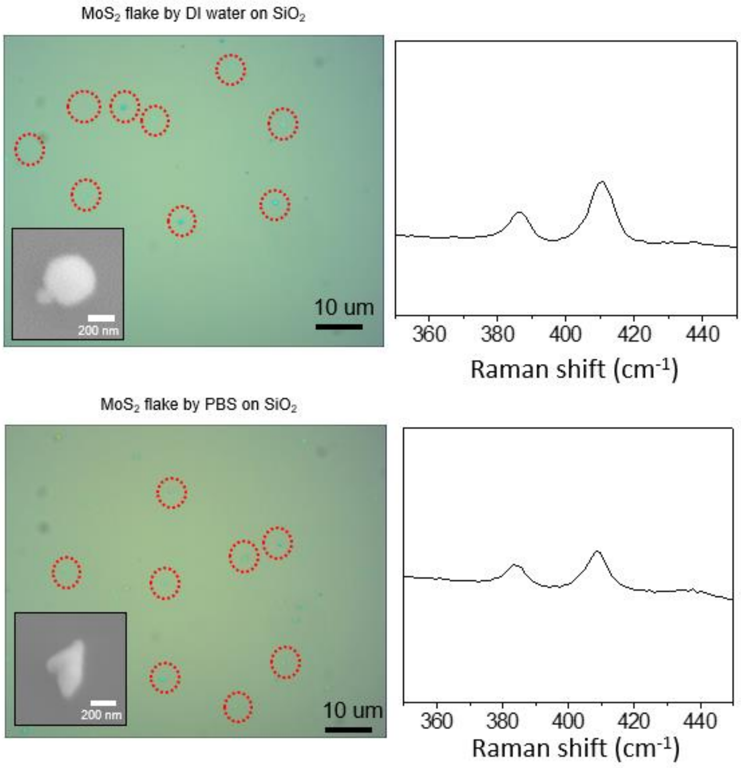

Supplementary Figure 11 | Morphological and structural characterization of MoS_2 flake obtained from the continuous film. OM images of dispersed MoS_2 flake in DI water and PBS solution and corresponding Raman spectra on SiO_2 substrate. Insets are the SEM image of the MoS_2 flakes in size of 300~400 nm.

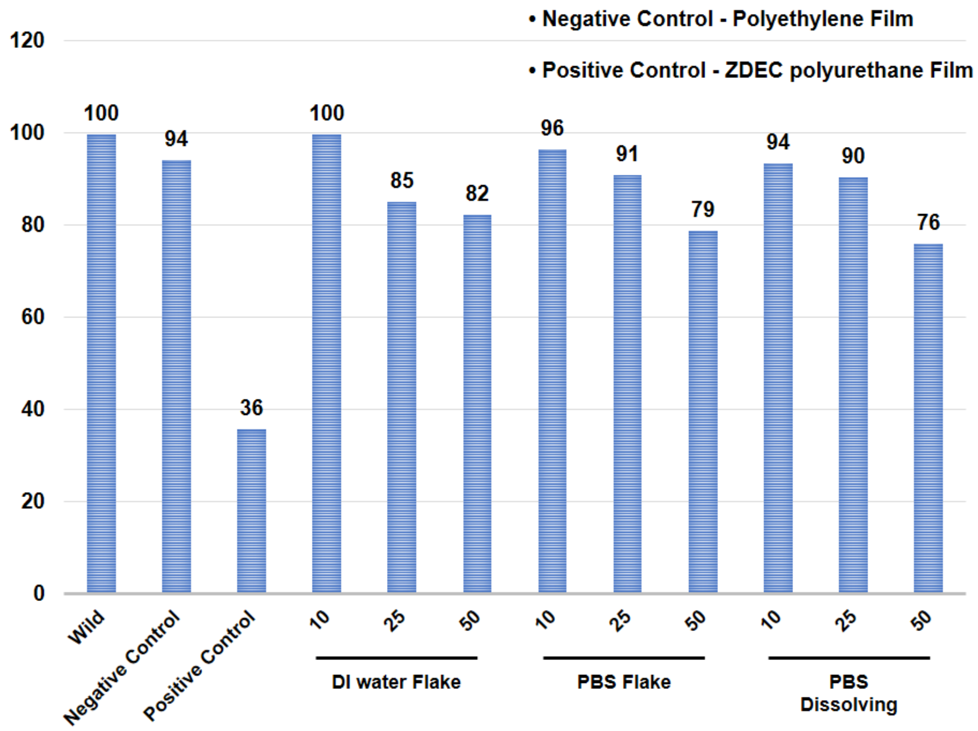

Supplementary Figure 12 | The statistical results of cell viability. Cell viability of L-929 cells on MoS_2 flake in DI water and PBS solution, dissolved MoS_2 in PBS, Culture medium, HDPE and ZDEC by CCK-8 assay.

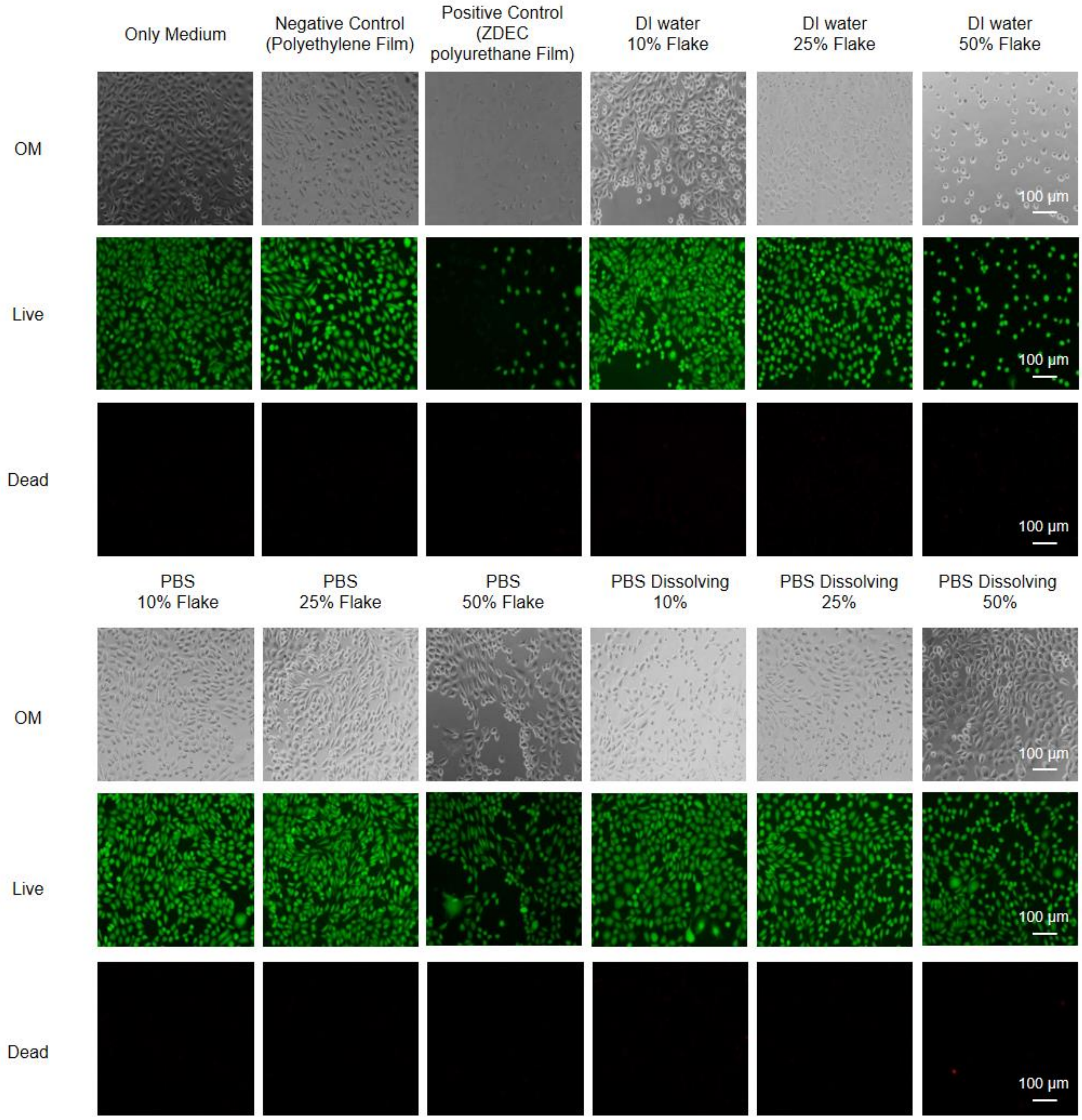

Supplementary Figure 13 | Cell culture results in different mediums. Set of differential interference contrast images and fluorescent images showing the cell viability of culture medium, HDPE, ZDEC, MoS_2 flake in DI water (10%, 25%, 50%), MoS_2 flake in PBS (10%, 25%, 50%), and dissolved MoS_2 in PBS (10%, 25%, 50%).

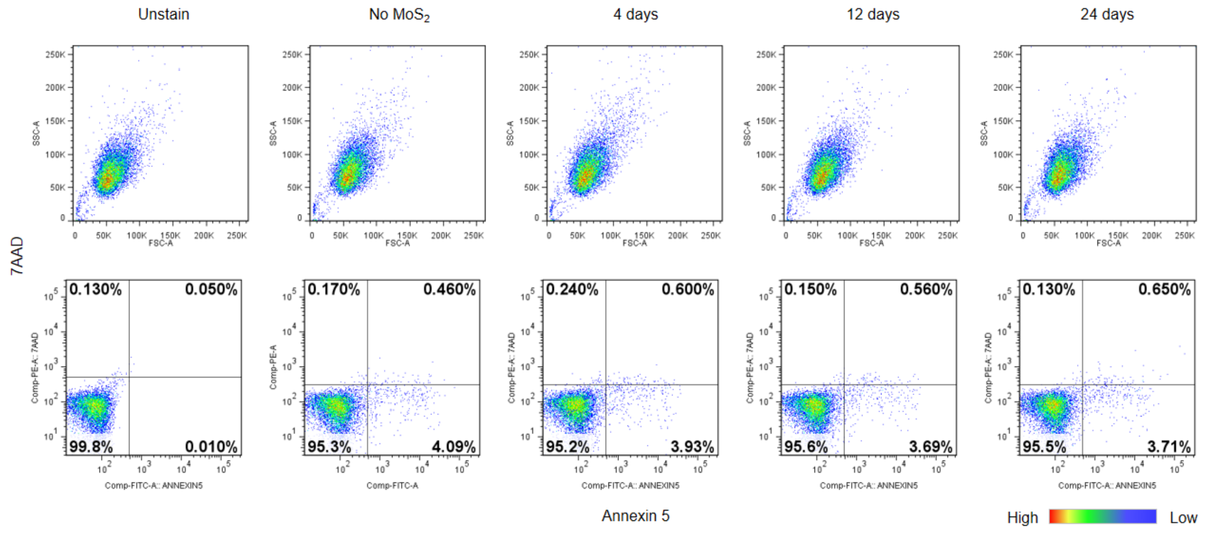

Supplementary Figure 14 | Apoptotic population study based on 2D MoS_2 film. Estimation of apoptotic populations in L929 of culture on MoS_2 film over 24 days using by Annexin V and 7-AAD, stained using flow cytometry.

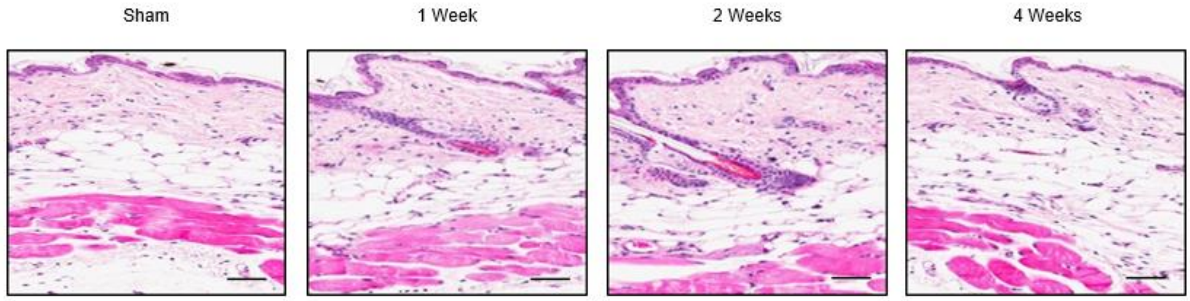

Supplementary Figure 15 | *In vivo* biocompatibility studies. Representative microscope images (scale bar: 100 μm) of hematoxylin and eosin (H&E) stained tissue sections after 0-4 weeks post-implantation.

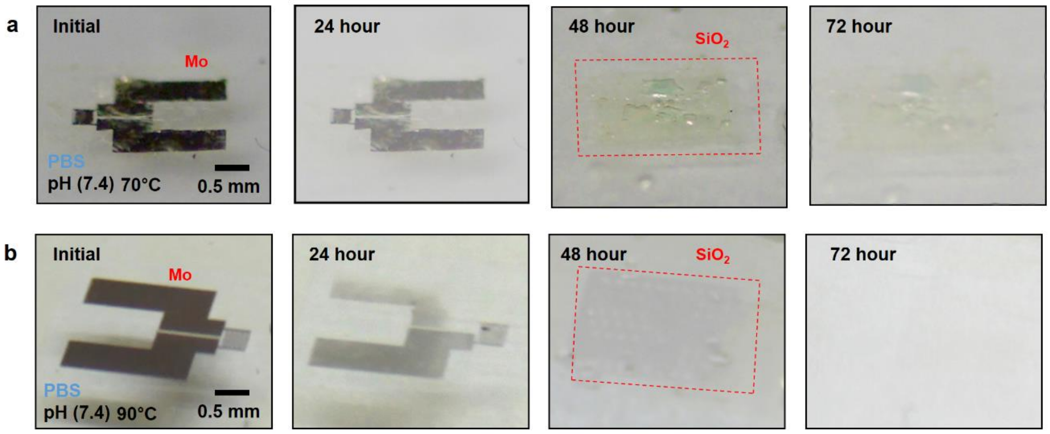

Supplementary Figure 16 | Time-dependent dissolution process of the 2D MoS_2 bioabsorbable device. Dissolution images collected at 4 stages of the accelerated condition of a system immersed in an aqueous buffer solution (pH 7.4) at 70 °C (**a**) and 90 °C (**b**).

Supplementary References:

1. Wang, X., Shen, X., Wang, Z., Yu, R. & Chen, L. Atomic-scale clarification of structural transition of MoS_2 upon sodium intercalation. *ACS nano* **8,** 11394-11400 (2014).
2. Zhang, L. *et al*. In situ TEM observing structural transitions of MoS_2 upon sodium insertion and extraction. *RSC Adv.* **6,** 96035-96038 (2016).
3. Wang, Z. *et al*. Chemical Dissolution Pathways of MoS_2 Nanosheets in Biological and Environmental Media. *Environ. Sci. Technol.* **50,** 7208-7217 (2016).
